# The Medical Student’s Vade-Mecum

**Published:** 1847-07

**Authors:** 


ART VIII.—*The Medical Student's Vade-Mecum*, or Examinations upon Anatomy, etc. etc. etc. Second edition, revised and greatly enlarged. By GEORGE MENDENHALL, M.D., etc. etc. Philadelphia: 1847.

This is another "help" to students, coming under the head of *multum in parvo*, and, no doubt, will help many a student through the "*green room.*"

